# Parental Educational Attainment Differentially Boosts School Performance of American Adolescents: Minorities’ Diminished Returns 

**Published:** 2019-03

**Authors:** Shervin Assari, Cleopatra H. Caldwell

**Affiliations:** 1 Department of Family Medicine, Charles R Drew University of Medicine and Science, Los Angeles, CA, USA; 2 Center for Research on Ethnicity, Culture and Health, School of Public Health, University of Michigan, Ann Arbor, MI, USA; 3 Department of Health Behavior and Health Education, School of Public Health, University of Michigan, Ann Arbor, MI, USA

**Keywords:** Educational Attainment, Race, Ethnicity, Blacks, Hispanics, Latinos, African Americans, Socioeconomic Status, School Performance

## Abstract

**Objective:** To explore racial and ethnic variation in the effects of parental educational attainment on students’ grade point average (GPA) in the US. As suggested by the Minorities' Diminished Returns (MDR) theory, socioeconomic status (SES) systemically results in smaller outcomes for non-Whites compared to Whites. We still know very little about diminished trans-generational returns of SES resources such as parental educational attainment. For example, the differential impacts of parental educational attainment on school performance of youth from various racial and ethnic backgrounds are still unknown.

**Materials and methods:** The Population Assessment of Tobacco and Health (PATH 2013 - 2014) is a nationally representative survey in the US. The total sample was 10,701 youth (12-17 years old) were enrolled. The independent variable was parental educational attainment. The main outcome was GPA measured using self-report. Age, gender, and parental marital status were the covariates. Race and ethnicity were the effect modifiers. Linear regression models were used to analyze the data.

**Results:** Overall, higher parental educational attainment was associated with a higher GPA, independent of all possible confounders. Race and ethnicity, however, both showed significant interaction with parental educational attainment on students’ GPA, indicating smaller positive effects of parental educational attainment on students’ GPA for Hispanic and Black compared to non-Hispanic White youth.

**Conclusion:** The boosting effect of parental educational attainment on GPA is smaller for Black and Hispanic compared to White youth. To minimize diminished returns of parental educational attainment for Black and Hispanic families, there is a need for innovative public and social policies and programs that are not limited to equalizing SES but also address the structural barriers that disproportionately limit upward social mobility of racial and ethnic minority students and their families. The US society should reduce extra costs of upward social mobility for racial and ethnic minority families. As the underlying mechanisms are multifaceted, multi-level approach is needed to undo minorities’ diminished returns, so every individual can gain the same tangible outcome from their SES resources.

## Introduction

Educational attainment, one of the main socioeconomic status (SES) indicators, is among the strongest social determinants of well-being (1-3). High educational attainment, both that of self and also of parent, is a strong predictor of developmental outcomes (2, 3). Families with high educational attainment are less likely to experience poverty, stress, problem behaviors, and poor health (4-7). Parental educational attainment, not; defined as the highest education level of parents, is a salient protective factor for a wide range of offspring outcome (4-7). At least some of the racial and ethnic disparities in youth outcomes are attributed to low parental educational attainment in racial and ethnic minority families (5, 8, 9).

Own educational attainment (10) as well as parental educational attainment (4, 11, 12), however, may not generate equal outcomes across various demographic and social groups. Members of the majority and minority groups may differently be able to navigate the social system and translate their human resources to tangible outcomes (12-17). As a result, the magnitude, direction, and the mechanism of the effects of own education and parental educational attainment widely vary for US sub-populations (13, 15, 18-20). 

Some empirical evidence, however, has suggested that own and parental educational attainment may better translate to desirable outcomes for non-Hispanic Whites than Hispanics and Blacks (13, 18). For example, effects of own educational attainment on smoking cigarette (21), drinking alcohol (22), healthy diet (23), obesity and overweight (20), depression (24), suicide (25), and mortality (26) are all smaller for Hispanics and Blacks than non-Hispanic Whites. Some studies even have linked high SES to poor mental health of Blacks (27,28). In some studies using national sample of Black youth (27) and adults (24), high SES positively correlated with depression and depressive symptoms. In one study, high educational attainment was a risk factor for suicidal ideation among Black women (25). These patterns may be in part because for racial and ethnic minorities, high SES also reflects more frequent contact with Whites in the workplace and school, which increases discriminatory experiences (29, 30). Discrimination itself is a risk factors for several poor developmental and health outcomes (31-33).

Although most of the evidence on Minorities’ Diminished Returns (MDRs) of SES are on own rather than parental educational attainment, some recent studies have shown similar MDRs for the trans generational effects of parental educational attainment on the outcomes of the offspring (15, 19, 20). In multiple studies (15, 19, 20), family SES particularly parental educational attainment showed larger effects on body mass index (BMI) (20), self-rated health (SRH) (19) attention deficit hyperactivity disorder (ADHD) (34), impulse control (15), and school attachment (35), for White than Black youth. In a recent study, parental educational attainment even better boosted educational attainment (11) and mental health (12) of White than Black youth. 

A part of the racial and ethnic inequalities in returns of parental educational attainment (MDRs) may be due to the systemic differences in the education quality or labor market discrimination which are parts of institutional and structural racism and colorism (14, 36). As society differently treats sub-populations, Blacks, Hispanics, and Whites differ in how they can mobilize their educational attainment to gain desired outcomes (13, 18). Non-Whites pay extra psychosocial costs for their upward social mobility than Whites (11, 37). Non-Whites also need to put additional efforts to climb the social ladder, in comparison to Whites (11, 37). Given the history of "slavery", "Jim Crow", and "residual racism" in terms of segregation and discrimination, educational attainment better help Whites than non-Whites to gain employment, social power, income, and purchase power that are all essential to secure desired outcomes (13, 18). As a result, the very same educational attainment brings more employment opportunities and better life conditions for Whites than Blacks and Hispanics (13, 18). Blacks and Hispanics may gain their education in inner city schools which are low in resources. Black and Hispanic children and youth are also frequently discriminated against inside (38) and outside (39) schools. Such discrimination increases risk of several health, behavioral, and developmental problems (31-33). Discrimination is one of the causes of MDRs of educational attainment (40, 41). All of these processes reduce the effects of own educational attainment as well as parental educational attainment on positive tangible outcomes for minorities such as Hispanics and Blacks compared to non-Hispanic Whites.

To better understand racial and ethnic differences in the trans-generational effects of parental educational attainment on school performance of American youth, we used nationally representative data to compare the effects of parental educational attainment on youths’ GPA by race and ethnicity.

## Materials and methods

***Design and settings:*** This is a secondary analysis of the Population Assessment of Tobacco and Health (PATH) youth data. PATH is funded by the NIH and FDA, and focuses on tobacco use of American adolescents and adults. From the 49,000 people 12+ years old who were enrolled to the PATH study at baseline, about 14,000 were youth (12-17 years old). Wave 1 data of the PATH study were collected between 2013 and 2014. For the purpose of this study, we used publicly available data set. Data were downloaded from the Inter-university Consortium for Political and Social Research (ICPSR) website. 

***Sample and sampling: ***The PATH youth study enrolled individuals who were 1) civilian, 2) non-institutionalized US population, and 3) 12-27 years of age. A four-stage stratified and clustered sampling strategy was used. First, 156 primary sampling units (PSUs) were selected. In PATH, PSUs are either a county or a group of counties. Second, smaller geographical segments in each PSU were selected. Third, residential addresses in those selected geographic segments were selected, using the US Postal Service data files. Fourth, one person was chosen from each sampled household. Analytical sample in this study was 10,701 12-17 year old youth.

***Study variables:*** The study variables include race, ethnicity, demographic factors (age and gender), family SES (parental educational attainment), marital status of the parents (family structure), and GPA. 

*Race.* Race was measured as self-identified. Race in the current study was a dichotomous moderator variable (White = 0, Black = 1). 

*Ethnicity.* Ethnicity was measured as self-identified. Ethnicity in the current study was a dichotomous moderator variable (non-Hispanic = 0, Hispanic = 1).

*Socioeconomic Status. *Parental educational attainment was a five-level variable as below: (1) Less than High School, (2) High school graduate or equivalent, (3) Some college including no degree or Associates degree, (4) Bachelor's degree, and (5) Advanced degree. This variable was treated as a continuous measure ranging from 1 to 5, with a higher score indicating higher family SES.

*Grade Point Average (GPA).* Participants were asked “What is your current overall GPA?” The answers were “9 = Mostly A's, 8 = A's and B's, 7 = Mostly B's, 6 = B's and C's, 5 = Mostly C's, 4 = C's and D's, 3 = Mostly D's, 2 = D's and F's, and 1 = Mostly F's.GPA was treated as an interval variable, with a range from 1 to 9, with a higher score indicating a better school performance. 

***Statistical Analysis:*** We used SPSS 23.0 statistical package for our data analysis. Frequencies as well as mean and standard deviation (SD) were reported to describe the sample. For multivariable analysis, we ran two linear regression models in the pooled sample. *Model 1* only included the main effects of parental educational attainment, race, ethnicity, and study covariates. *Model 2* also included the race, ethnicity by parental educational attainment interaction terms. In both models, GPA was the outcome variable, and parental educational attainment was the predictor. Gender, age, and marital status of the family were covariates. Regression coefficients (b), 95% Confidence Intervals (CIs), SE, as well as p values were reported. 

***Ethics: ***The PATH study protocol is approved by the West at Institutional Review Board (IRB). All participants gave a written assent. All the guardians and parents signed an informed consent.

## Results

***Univariate Analysis*** *:* This analysis included 10,701 youth. Table 1 describes the pooled sample.About 19% were Blacks and 22% were Hispanic. The sample was slightly more boys (52%) than girls (48%) (Table 1).

**Table 1:** Descriptive statistics

	**n**	**%**
Race		
White	8,678	81.1
Black	2,023	18.9
Ethnicity*^ a^		
Non-Hispanic	8,179	77.8
Hispanic	2,329	22.2
Age		
12-15	5,474	51.2
16-17	5,227	48.8
Gender		
Women	5,143	48.2
Men	5,531	51.8
Marital Status*^ a^		
Not Married	3,870	36.2
Married	6,817	63.8
	**Mean**	**SD**
Parental Educational Attainment (1-5)*^ b^	2.84	1.22
GPA *^b^	7.29	1.64

GPA: Grade Point Average

**Table 2:** Bivariate correlations in the overall sample

	**1**	**2**	**3**	**4**	**5**	**6**	**7**
1 Race (Black)	1.00	-.14^b^	.00	.00	-.25 b	-.09 b	-.14 b
2 Ethnicity (Hispanic)		1.00	.00	-.04^b^	-.04 b	-.28 b	-.06 b
3 Gender (Male)			1.00	0.01	.00	.01	-.18 b
4 Age (Years)				1.00	.00	-.01	-.08 b
5 Marital Status (Married)					1.00	.19 b	.18 b
6 Educational Attainment (Family SES; 1-5)						1.00	.23 b
7 Grade Point Average (GPA)							1

a p < 0.05

SES: Socioeconomic Status

Pearson Correlation Test

b p < 0.01

***Bivariate Analysis:*** Race, ethnicity, parental marital status, and parental educational attainment were correlated with number of GPA (Table 2).

***Multivariate Analysis*** *:* Table 3 shows the results of the two linear regression models, both in the overall sample. *Model 1 *(Main Effect Model) showed a positive effect of parental educational attainment on GPA. *Model 2 *(Interaction Model) showed interactions between race and ethnicity with parental educational attainment on GPA, suggesting weaker boosting effects of parental educational attainment on GPA for Hispanic and Black youth compared to their non-Hispanic White counterparts (Table 3).

## Discussion

We found an overall positive effect of parental educational attainment on GPA among American youth. We, however, found evidence suggesting racial and ethnic variations in the boosting effect of parental educational attainment on youth GPA. The GPA gain from high parental educational attainment was significantly smaller for Hispanic and Black than larger for non-Hispanic White youth. 

These findings are similar to what the Literature has already show regarding the MDRs of SES indicators for Blacks and Hispanics than non-Hispanic Whites (10, 14, 42, 43). Such effects are documented within individuals, within families, and across generations. MDRs are repeatedly shown across age groups, SES resources, populations, cohorts, and outcomes (13, 18). Although most of the MDRs literature is focused on individuals, trans-generational MDRs suggest that parental educational attainment do not similarly generate outcomes across racial and ethnic groups (15, 19, 20). 

**Table 3:** Summary of two linear regressions by race / ethnicity

	**B**	**SE**	**Beta**	**95% CI**		**P**
Model 1						
Race (Black)	-0.41	0.04	-0.10	-0.49	-0.33	.000
Ethnicity (Hispanic)	-0.04	0.04	-0.01	-0.11	0.03	.295
Married	0.39	0.03	0.12	0.33	0.46	.000
Gender (Boy)	-0.58	0.03	-0.18	-0.64	-0.52	.000
Age	-0.23	0.03	-0.07	-0.28	-0.17	.000
Parental Educational Attainment (1-5)	0.26	0.01	0.20	0.24	0.29	.000
Constant	6.79	0.05		6.68	6.89	.000
Model 2						
Race (Black)	0.07	0.10	0.02	-0.12	0.27	.465
Ethnicity (Hispanic)	0.45	0.08	0.11	0.28	0.61	.000
Married	0.39	0.03	0.11	0.32	0.45	.000
Gender (Boy)	-0.58	0.03	-0.18	-0.64	-0.52	.000
Age	-0.22	0.03	-0.07	-0.28	-0.16	.000
Parental Educational Attainment (1-5)	0.34	0.02	0.25	0.31	0.37	.000
Parental Educational Attainment (1-5) × Race	-0.17	0.03	-0.12	-0.24	-0.11	.000
Parental Educational Attainment (1-5) × Ethnicity	-0.19	0.03	-0.13	-0.25	-0.13	.000
Constant	6.55	0.06		6.43	6.67	.000

B: Unstandardized regression coefficients; Beta: Standardized regression coefficient; SE: Standard Error; CI: Confidence Interval

We should not blame Hispanics and Blacks for the observed MDRs. MDRs are attributed to the existing racism and colorism in the American social structure. The US social and political system has failed Black and Hispanic families by charging them extra costs for their upward social mobility. Upward social mobility is not similarly easy for non-Hispanic Whites and non-Hispanic Blacks (13, 18). Historically, Whites have had the highest political and social power. As a result, policies have historically maximized the gains of the majority groups (13, 18). 

Highly educated Hispanic and Black families face disproportionately higher levels of barriers in their daily lives. Such barriers reduce Hispanic and Black families’ chance of gaining outcomes from their educational attainment. Highly educated Black and Hispanic families frequently experience discrimination on a daily basis. Discrimination reduces the gains that are expected to follow educational attainment (39, 44, 45). In US, a race–and color aware society, people are treated based on their social groups, skin color, and race rather than their potentials. As a result, highly educated Black and Hispanic families do not access the same opportunity structure compare to their White counterparts.

Minorities’ diminished returns (MDRs) (13, 18) refer to smaller gains of SES resources on outcomes in socially marginalized groups. Similar results are shown across age groups, SES resources, and outcomes. In all of these studies, educational attainment is followed by a smaller impact for Hispanics and Blacks than non-Hispanic Whites. Educational attainment better promotes various outcomes for youth (15, 19, 20), adults(14), and older adults (22) for Whites than Hispanics and Blacks. MDRs do not only apply to educational attainment (10) but also employment (46), income and marital status (16). 

***Limitations:*** This study is not without methodological limitations. First, cross-sectional studies do not allow causal inferences. Longitudinal studies with multiple observations of GPA over time are needed to shown any causal links between race, ethnicity, parental educational attainment, and GPA. Similar to the rest of the literature on MDRs, this study exclusively focused on Hispanics, Blacks, and Whites. We need more studies on other racial and ethnic groups. MDRs are not limited to race and ethnicity. Any marginalizing identity may alter the gains of educational attainment on outcomes. This study only focused on MDRs of one SES indicator. There is a need for studies on MDRs of wealth, employment, health, and marital staus. More research is needed on mediating and moderating effects of employment conditions, stress, discrimination, and behaviors on the observed patterns in this study. Despite these listed limitations, the results of the current study still extend the literature on trans-generational diminished returns of SES in marginalized groups.

## Conclusion

Compared to their non-Hispanic White counterparts, Hispanic and Black youth gain less GPA from their parental educational attainment.
